# Polyol pathway-generated fructose is indispensable for growth and survival of non-small cell lung cancer

**DOI:** 10.1038/s41418-024-01415-1

**Published:** 2024-11-20

**Authors:** Annemarie Schwab, Mohammad Aarif Siddiqui, Vignesh Ramesh, Paradesi Naidu Gollavilli, Adriana Martinez Turtos, Sarah Søgaard Møller, Luisa Pinna, Jesper F. Havelund, Anne Mette A. Rømer, Pelin Gülizar Ersan, Beatrice Parma, Sabine Marschall, Katja Dettmer, Mohammed Alhusayan, Pietro Bertoglio, Giulia Querzoli, Dirk Mielenz, Ozgur Sahin, Nils J. Færgeman, Irfan A. Asangani, Paolo Ceppi

**Affiliations:** 1 Interdisciplinary Center for Clinical Research (IZKF), Friedrich-Alexander University of Erlangen-Nuremberg, Erlangen, Germany; 2 Experimental Medicine 1, Friedrich-Alexander University of Erlangen-Nuremberg, Erlangen, Germany; 3 Department of Biochemistry and Molecular Biology, University of Southern Denmark, Odense, Denmark; 4 Biotech Research and Innovation Centre (BRIC), University of Copenhagen, Copenhagen, Denmark; 5 Department of Molecular Biology and Genetics, Faculty of Science, Bilkent University, Ankara, Turkey; 6 Laboratory of Immunobiology, Université Libre de Bruxelles- Faculty of Science, Brussels, Belgium; 7 Institute of Functional Genomics, University of Regensburg, Regensburg, Germany; 8 Perelman School of Medicine, University of Pennsylvania, Philadelphia, PA USA; 9 Department of Bioenergetics & Neurometabolism, Dasman Diabetes Institute, Dasman, Kuwait; 10 Division of Thoracic Surgery, IRCCS Azienda Ospedaliero Universitaria di Bologna, Bologna, Italy; 11 Pathology Unit, IRCCS Azienda Ospedaliero Universitaria di Bologna, Bologna, Italy; 12 Ospedale Sacro Cuore Don Calabria, Verona, Italy; 13 Division of Molecular Immunology, Department of Internal Medicine 3, Friedrich-Alexander Universität Erlangen-Nürnberg and Universitätsklinikum Erlangen, Nikolaus-Fiebiger-Center, Erlangen, Germany; 14 Department of Biochemistry & Molecular Biology – College of Medicine, Hollings Cancer Center, Medical University of South Carolina, Charleston, SC USA

**Keywords:** Non-small-cell lung cancer, Cancer metabolism

## Abstract

Despite recent treatment advances, non-small cell lung cancer (NSCLC) remains one of the leading causes of cancer-related deaths worldwide, and therefore it necessitates the exploration of new therapy options. One commonly shared feature of malignant cells is their ability to hijack metabolic pathways to confer survival or proliferation. In this study, we highlight the importance of the polyol pathway (PP) in NSCLC metabolism. This pathway is solely responsible for metabolizing glucose to fructose based on the enzymatic activity of aldose reductase (AKR1B1) and sorbitol dehydrogenase (SORD). Via genetic and pharmacological manipulations, we reveal that PP activity is indispensable for NSCLC growth and survival in vitro and in murine xenograft models. Mechanistically, PP deficiency provokes multifactorial deficits, ranging from energetic breakdown and DNA damage, that ultimately trigger the induction of apoptosis. At the molecular level, this process is driven by pro-apoptotic JNK signaling and concomitant upregulation of the transcription factors c-Jun and ATF3. Moreover, we show that fructose, the PP end-product, as well as other non-glycolytic hexoses confer survival to cancer cells and resistance against chemotherapy via sustained NF-κB activity as well as an oxidative switch in metabolism. Given the detrimental consequence of PP gene targeting on growth and survival, we propose PP pathway interference as a viable therapeutic approach against NSCLC.

## Introduction

Non-small cell lung cancer (NSCLC) is one of the most common cancer entities. Although numerous improvements have been made in the fields of immuno- or targeted therapy, NSCLC is still leading the statistics with the highest cancer-related mortality worldwide, highlighting the urgent need to identify new therapy options for improving patient outcome. Two of the major obstacles impeding patient survival are metastasis formation and therapy resistance, ultimately leading to patient relapse and death [1]. A critical regulator of both processes is the highly dynamic program of the epithelial-to-mesenchymal transition (EMT) which enables cancer cells with a strongly invasive and drug-resistant phenotype [2, 3]. We have recently reported a metabolic pathway, the polyol pathway (PP), and uncovered its connection with EMT in cancer [4]. This pathway is responsible for the conversion of glucose into fructose, which is carried out through two enzymatic reactions catalyzed by aldose reductase (AKR1B1) and sorbitol dehydrogenase (SORD) [5]. While its activity under normoglycemic conditions is considered negligible [6, 7], glycolytic flux through the PP is known to rapidly increase in a hyperglycemic environment, such as diabetes. In this context, PP activity has been extensively linked to diabetic complications caused by increased reactive oxygen species (ROS) [8]. Given the known glucose dependency of cancer, known as the Warburg effect, as well as the role of oxidative stress-induced inflammation during carcinogenesis, PP can be regarded as an excellent target to study in the context of cancer. To date, it has been shown that impairing the function of the rate-limiting enzyme of PP, AKR1B1 diminishes cell growth, formation of precancerous lesions, migratory capabilities as well as metastasis formation [9, 10]. Mechanistically, these functions were connected to the regulation of the EMT program and ROS-induced inflammatory responses. However, most cancer-related studies have so far been limited to exploring the role of AKR1B1 alone, owing to its broad substrate affinity and unique PP-independent functions, most importantly in the synthesis of prostaglandins and other hormone precursors [11, 12]. We have subsequently demonstrated the previously underestimated second PP enzyme, SORD, to be equally essential for EMT, cancer cell growth and stem cell properties, implicating for the first time that the metabolic activity of the PP is important for carcinogenesis [4].

In this study, using different lung adeno- and squamous carcinoma models, we uncover both PP enzymes to be indispensable for NSCLC growth and survival. Mechanistically, PP deficiency provokes multifactorial deficits, ranging from energetic breakdown to DNA damage, ultimately inducing apoptosis and cell death. At the molecular level, we could demonstrate the involvement of JNK signaling and upregulation of AP-1 family of transcription factors. Via several rescue experiments, we establish the PP end-product fructose as an important fuel for lung cancer survival and therapy resistance. We unveil fructose importance for NSCLC pathogenesis and propose PP interference as a promising therapeutical approach against lung cancer.

## Methods

### Cell culture

A549, NCI-H596 (adenocarcinoma), Calu1, SK-MES-1, NCI-H520 (squamous cell carcinoma), NCI-H460 and NCI-H1299 (large cell carcinoma) were purchased from ATCC. A549 and HEK-293T cells were maintained in DMEM, while all other cells were maintained in RPMI-1640, supplemented with 10% fetal bovine serum (FBS) and 1% penicillin/streptomycin and incubated at 37 °C and 5% CO_2_. All cell lines were regularly tested for mycoplasma contamination and authenticated by STR profiling.

### Lentiviral production and transduction

Lentiviral particles were produced by transfecting HEK-293T cells with the third-generation packaging system consisting of pMDL, VSVg, and Res-Rev plasmids (2 µg) together with 8 µg of control, knockdown or overexpression vectors. MISSION^©^ shRNA plasmids (Sigma) were used for stable knockdown. Sequences TRCN0000288738, TRCN0000288741 and TRCN0000288812 were used for knocking down AKR1B1, whereas SORD was knocked down using the sequences TRCN0000028052, TRCN0000028069, and TRCN0000028106. Overexpression was achieved with OmicsLink™ Expression-ready ORF cDNA vectors (Genecopoeia, C0237). Following 2 days of virus multiplication, the supernatant was used to transduce target cells using the spin-infection method at 700 rpm for 45 min in the presence of 8 µg/ml polybrene. Two days after transduction, positive cells were selected using 3 µg/ml puromycin. The packaging vectors were shared from Prof. Dr. Med. Beate Winner, Stem Cell Biology, University Clinic Erlangen.

### Life-cell proliferation and death assays

To determine growth and viability, cells were plated in 4–6 replicates at a density of 5000 cells per well in 96-well plates. Images were recorded every 2–4 h using the IncuCyte Zoom Live Cell Imaging System (Sartorius). As a readout of proliferation, cells with a stable expression of fluorescent histone marker (H2B-RFP) were used to count the number of nuclei (# of nuclei/mm^2^). Alternatively, the percentage of occupied area (% of confluence) was measured over time. To determine cell death, cytotox green reagent (Sartorius) was added at a final dilution of 1:5000, whereas caspase 3/7 green reagent (Sartorius) was used at a final concentration of 5 μM (1:1000) for apoptosis measurement. In both cases, the number of individual green-fluorescent signals were counted over time (CyTox/mm^2^ or Casp3/7/mm^2^).

### Compound treatments

To assess cytotoxicity of chemotherapeutic drugs or inhibitors, cells were plated in normal media in 96-well plates at a density of 4000–6000 cells per well and 4–5 replicates were used per condition. After cell attachment, media was replaced containing either cisplatin (CDDP, Santa Cruz), pemetrexed (PTX, Tocris Bioscience), N-Acetyl-l-cysteine (NAC, Sigma), 2-deoxy-d-glucose (2-DG, sc-202010) or an adequate vehicle control. Cytotoxic effects and proliferation were assessed for 3–5 days using the methods described above.

To determine the effects of NF-κB activity inhibition, 5000 cells per well maintained in 5 mM glucose, fructose or galactose were treated with a serial drug dilution of R7050 or SM7368. After 40 h of drug treatment, cell confluence was determined using the IncuCyte Zoom Live Cell Imaging System. Confluence values of treated cells were normalized to the control group (100%) and log-transformed using GraphPad Prism. To determine the half maximal inhibitory concentration (IC_50_), a nonlinear regression (curve fit) was applied using the equation log(inhibitor) vs. normalized response.

### Protein isolation and quantification

Proteins from adherent cells were isolated in RIPA buffer supplemented with Halt protease & phosphatase inhibitor cocktail (both Thermo) and quantified using the Pierce BCA protein assay kit (Thermo) according to the manufacturer’s instructions. Ten to twenty micrograms protein per sample were used to perform SDS-PAGE, followed by blotting onto PVDF membranes (Thermo). After blocking in 5% BSA in TBS-T buffer, primary antibodies were incubated overnight at 4 °C, followed by staining with secondary HRP-conjugated antibodies (Southern Biotech). Protein bands were visualized using the Pierce ECL western blotting substrate (Thermo), X-ray CL-XPosure films (Thermo) and the automatic film processor CP1000 (AGFA). Protein band quantification was performed using ImageJ. Antibodies were AKR1B1 (Thermo, PA5-12316), SORD (Thermo, PA5-37390), β-ACTIN (Cell Signaling, 12262), γH2AX (Cell Signaling, 2577), AKT (Cell Signaling, 2938), ATF3 (Sigma, HPA001562), c-Jun (Cell Signaling, 9165), cleaved PARP (Cell Signaling, 9541), p65 (Cell Signaling, 8242), p-AKT (S473) (Cell Signaling, 4060), p-JNK (Cell Signaling, 4668) and p-p65 (Cell Signaling, 3033). All secondary HRP-conjugated antibodies were from Southern Biotech. β-ACTIN was used as a loading control.

### NF-κB luciferase reporter assay

To determine NF-κB activity, control, shAKR1B1 or shSORD cells were plated at a density of 12,000 cell per well and transfected the following day with the NF-κB luciferase reporter vector (BPS Bioscience, #60614) using Lipofectamine^TM^ 2000 according to the manufacturer’s instructions. Activities of both Renilla and Firefly luciferase reporter were measured using the Dual-Glo^®^ Luciferase System (Promega). Background signals from non-transfected controls were subtracted from all luminescence measurements of both luciferases. The ratio of Renilla/Firefly served as normalization of NF-κB activity to a reporter housekeeping control, accounting for transfection efficacy differences. Ratios for pLKO control (Glc) cells were set as baseline 1.

### Measurement of oxidative stress

As an indicator of general oxidative stress, cells were stained with CM-H_2_DCFDA (Thermo Fisher Scientific) at a final concentration of 3.12 µg/ml in complete culture media for 30 min at 37 °C and 5% CO_2_. Fluorescence signals were recorded using the CytoFLEX flow cytometer (Beckman Coulter) and analyzed with FlowJo (V10.1). Alive cells were gated on SSC-A vs FSC-A, doublets were excluded using FSC-A vs FSC-H and CM-H_2_DCFDA positivity was analyzed using the FITC channel. Unstained samples of control, knockdown or treatment conditions were used to determine background fluorescence and appropriate gate setting.

### Gas chromatography–mass spectrometry (GC-MS)

For measuring intracellular fructose levels, 500,000 cells per sample were plated and isolated the following day in 80% ice-cold methanol. Samples were vortexed and spiked with 10 μl internal standard containing [U-^13^C_6_]-Fructose at a concentration of 1 mM. Samples were centrifuged at 9560 × *g* and 4 °C for 6 min and supernatants were removed. The cell pellet was washed twice with 80% methanol, the supernatants were combined and dried in a vacuum evaporator (CombiDancer, Hettich AG, Bäch, Switzerland). Sugars were analyzed by GC-MS after methoximation and silylation using the derivatization protocol and instrumental setup as described [13]. An injection volume of 1 μl and splitless injection were applied. Intracellular metabolites were quantified using calibration curves with [U-^13^C_6_]-Fructose as internal standard and normalized to protein amount. Proteins were quantified by resuspending the pellet obtained after extraction in NaH_2_PO_4_ buffer (20 mM with 1.2% SDS), followed by the use of FluoroProfile Protein Quantification Kit (Sigma) according to the manufacturer’s instruction.

### Liquid chromatography–mass spectrometry (LC–MS)

To quantify the in vitro metabolic flux of the polyol pathway, [U-^13^C_6_]-Glucose tracing was performed in A549 and H1299 cells. Briefly, plko and shAKR1B1 cells were generated by seeding 100,000 cells a day before virus transduction. Cells were infected with 1/3 of the viruses to avoid massive cell death. Twenty-four hours post virus infection, cells were washed and 250,000 cells were seeded in six-well plates. Sixteen hours post cell seeding, 10 mM [U-^12^C_6_]-Glucose or [U-^13^C_6_]-Glucose-containing DMEM was added to the cells for 6 h. At endpoint, cells were collected in an extraction solvent containing methanol/acetonitrile/water (5:3:2 v/v/v) and centrifuge at 21,000 × *g* at 4 °C for 15 min. Supernatant were collected and stored at −80 °C. For UPLC–MS analysis, samples were lyophilized and resuspended in 25 µl of 80% acetonitrile. Three microliters were injected using a Vanquish Horizon UPLC system (Thermo Fisher Scientific) equipped with a Luna Omega Sugar column (2.1 × 150 mm, 100 Å, 3 µm, Phenomenex), maintained at 40 °C. Analytes were eluted at a flow rate of 300 µl/min using eluent A (water) and eluent B (acetonitrile). The gradient profile was as follows: 80% B from 0 to 2 min, 80–30% B from 2 to 6 min, 30% B from 6 to 6.5 min, and 30–80% B from 6.5 to 7 min followed by a 5-min equilibration period. The UPLC was coupled to a Bruker TimsTOF Pro 2 operated in negative ion mode. Data of intracellular glucose and fructose isotopologues were extracted and corrected for natural abundance using TASQ software (Bruker, version 2024B).

### Seahorse flux analysis

Bioenergetics of control and knockdown cell lines cultured under the indicated conditions were determined 5 days after lentiviral transduction using the XFe96 Extracellular Flux Analyzer (Seahorse Bioscience/Agilent Technologies, North Billerica, MA). Briefly, one day prior to the assay, cells were seeded in specialized cell culture microplates at a density of 12,000 cells per well using 6 replicates per condition. To perform the mitochondrial stress test, cells were incubated in XF base medium supplemented with 10 mM glucose, 2 mM l-glutamine and 1 mM sodium pyruvate (pH 7.4) for 1 h at 37 °C in a CO_2_-free environment. Extracellular acidification rate (ECAR) and oxidative consumption rate (OCR) values were recorded under baseline conditions and subsequently after injection of 1.2 µM oligomycin, 1.5 µM carbonyl cyanide-p-trifluoromethoxyphenylhydrazone (FCCP) and 1 µM rotentone/antimycin A.

### In vivo experiments

NSG mice (Jackson Laboratories) were used for xenograft experiments testing tumor growth of AKR1B1 knockdown cells. Briefly, plko control or shAKR1B1 cells at 5 days post-transduction were resuspended in 0.9% saline solution and mixed with Matrigel (Corning) at a ratio of 1:1 and 500,000 cells were injected into the right flanks of 8-12 week old female mice, using eight mice per condition. Tumor length and width were measured twice a week using a digital caliper and tumor volumes were calculated with the formula (length × width^2^)/2. Mice were sacrificed 30–35 days after injection, tumors were excised and weighed. Animal protocols were approved by the Institutional Animal Care and Use Committee of the Regierung von Unterfranken (NSG model). The maximum allowed size of 1766 mm^3^ was not achieved in any of these experiments.

To determine the effect of epalrestat treatment on tumor growth, 500,000 A549 cells were prepared in 100 μl of 1:1 mix of PBS and Matrigel (Corning) and injected into flank region of male nude mice, 6 per condition. Mouse weight and tumor volume were measured every second day using calipers. Tumor volumes were calculated as (length x width^2^) /2. Once the tumor volume had reached 80-100 mm^3^, xenografts were randomized into groups. Animals were treated with vehicle (5% DMSO, 30% PG, 65% PEG400) or epalrestat (EPR, 50 mg/kg) by daily oral gavage. EPR (CAS No. 82159-09-9) was obtained from Sigma-Aldrich and dissolved first in DMSO and heated at 50 °C for 10 minutes. 30% PG and 65% PEG400 were added individually and in order. The sample size for each experiment was selected based on measurements from previous similar experiments. All animal experiments were approved by the Animal Ethics Committee of Bilkent University. The studies were carried out in compliance with the ARRIVE guidelines.

### Study cohort

The study cohort consisted of 97 patients with primary NSCLC who had undergone surgical resection at the Sacro Cuore Don Calabria Hospital of Negrar, Verona (VR) between 2007 and 2017. Eighty-nine samples had sufficient slides and paraffin-embedded tissue blocks available. None of the patients received therapy before surgery. Tumors were classified according to the 2014 WHO classification and staging was done using the TMN staging manual (8^th^ edition). Patients’ demographics and clinical data were retrieved from the digital archives. Investigations have been conducted according to the principles expressed in the Declaration of Helsinki.

### Tissue microarray construction

For every case, all H&E stained were reviewed for diagnosis confirmation; one block was selected for tissue microarray (TMA) construction. For each block, five cores with a diameter of 1 mm were obtained from diverse areas of the tumor and randomly numbered from 1 to 5.

### Immunohistochemistry and scoring

From each block 4 µm sections were cut and stained with antibodies against AKR1B1 (Abcam, dilution 1:100) and SORD1 (Thermo, diluition 1:200). An OptiView DAB ICH Detection Kit (Ventana) was used according to the manufacture’s recommendations for the visualization of the primary anti-AKR1B1 and SORD1 antibodies. Stained sections were scanned using Ventana iScan HT and immunoreactivity scorings were evaluated by a pathologist. Intensity of staining (0–3+) and the percentage of stained tumor cells (0–100%) were used for calculation of IHC score. The IHC score was calculated by multiplying the staining intensity by the average percentage of stained tumor cells. We considered as adequate the scores showing a neoplastic component greater or equal to 30%. Alveolar macrophages were used as an internal control to validate the adequacy of AKR1B1 staining reaction. See Supplementary Fig. 1 for patient details.

### RNA sequencing and gene set enrichment analysis

RNA from A549 shAKR1B1 and pLKO control cells, cultivated under glucose or fructose growth conditions, was isolated using miRNeasy kit (Qiagen) following the manufacturer’s instructions, including a DNA digestion step. RNA-Seq libraries were constructed using the TruSeq sample Prep Kit V2 (Illumina). Briefly, 1–2 μg of purified RNA were poly-A selected and fragmented using a fragmentation enzyme. A template of poly-A selected/fragmented RNA was used for first- and second-strand synthesis. Subsequent procedures including end-repair to PCR amplification were performed in accordance with the library construction steps. Libraries were purified and validated for appropriate size on a 2100 Bioanalyzer High Sensitivity DNA chip (Agilent Technologies.) The DNA library quantification was performed using Qubit, followed by normalization to 4 nM. Libraries pooling was done in an equimolar fashion and diluted to 10 pM. Library pools were clustered and run on Nextseq500 platform with paired end reads of 75 bases, according to the manufacturer’s recommended protocol (Illumina). RNA-Seq reads were subjected to quality control analysis using FastQC v0.11.9 and aligned against human reference genome with GRCh38 build (release 102) with the respective Ensembl GTF annotation file using STAR v2.7.7a aligner. Read summarization was carried out using R 3.5.0 with featureCounts function in Rsubread package. With the obtained counts, differential gene expression analysis based on the negative binomial distribution was performed using DESeq2 package in R 3.5.0 and identified up- or downregulated genes between conditions with adjusted *p*-value < 0.01 and fold change of 2. Overlap analysis of the genes between conditions was performed using Venny 2.1 (https://bioinfogp.cnb.csic.es/tools/venny/). Gene set enrichment analysis was performed with the online application available at http://software.broadinstitute.org/gsea/index.jsp. Data are deposited in the GEO database with the accession number GSE214505.

### Statistical analysis

All statistical analyses were performed using GraphPad Prism 8.3.0, unless stated otherwise. Xenograft tumor growth was analyzed using multiple *t* tests with the Holm–Sidak method to correct for multiple comparisons. For comparison of more than one knockdown or treatment condition, the one-way ANOVA was applied with correction for multiple comparisons using the Dunnett (comparison to pLKO or control group) or Tukey method (comparison between all groups). A two-tailed student’s *t*-test was used to compare the statistical significance of the two groups (parametric). *P* values of statistical significance are illustrated as follows: *<0.05; **p < 0.01; ***p < 0.001; ****p < 0.0001, n.s. represents non-significant differences.

## Results

### Polyol pathway activity is essential for NSCLC pathogenesis

**Fig. 1:**
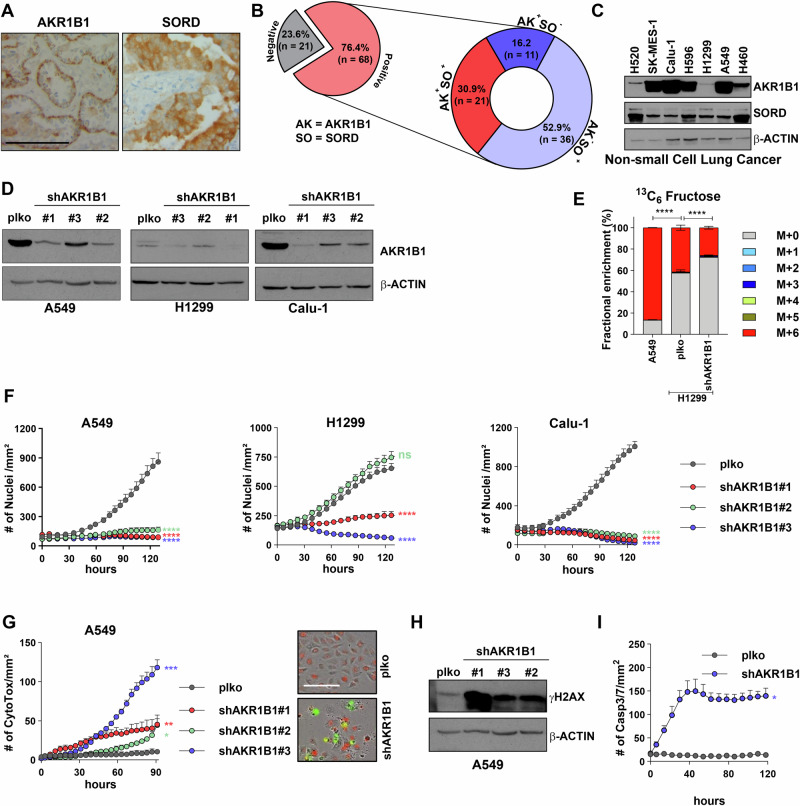
Knockdown of AKR1B1 results in growth arrest in vitro. **A** Representative images of AKR1B1 and SORD immunohistochemistry staining of NSCLC patient tissue samples (*n* = 89). The scale bar represents 100 µm. **B** Proportion of AKR1B1^-^, SORD^-^ single or double positive in human NSCLC specimens. **C** Western blot of AKR1B1 and SORD in a panel of established NSCLC cell lines. **D** Western blot of AKR1B1 in A549, H1299 and Calu-1 scrambled plko (control) cells and AKR1B1 knockdown cells transduced with three independent shRNA sequences. **E** Fractional enrichment of fructose in A549 plko cells, H1299 plko and shAKR1B1 cells upon [U-^13^C_6_]-Glucose tracing. P-values indicate significant differences among M + 6 isotopologues. **F** Real-time proliferation of plko control and three different shAKR1B1 knockdown in A549 (ADC), H1299 (LCC) and Calu-1 (SCC) cells. Proliferation of H2B-RFP positive cells was determined via quantification of fluorescent nuclei counts over time by the real-time imaging system Incucyte. **G** Cytotoxic effect of shAKR1B1 in A549 cells compared to plko control cells as quantified by the fluorescence of a green CytoTox dye in dead cells. Representative images with green-fluorescent signals depict the occurrence of cell death upon AKR1B1 knockdown. Scale bar is 150 µm. **H** Western blot of γH2AX in plko and three shAKR1B1 A549 cells. **I** Caspase 3/7-dependent apoptosis assay measuring activated caspase 3/7 in plko control and shAKR1B1 A549 cells measured by real-time imaging. Statistical tests are two-way ANOVA, Dunnett’s method except for (**E**) where it is *T*-test.

To assess polyol pathway activity in NSCLC, we first determined expression levels of both PP enzymes in a panel of NSCLC patients by immunohistochemical staining (Fig. 1A, Supplementary Fig. 1A, B). 76.4% of tissue samples revealed positivity for at least one PP enzyme (Fig. 1B). Among those, 30.9% were tested positive for both AKR1B1 and SORD expression, pointing towards a strong clinical relevance of PP activity in NSCLC with no prevalence for adeno- or squamous cell carcinoma subtype (Supplementary Fig. 1C, D). At the RNA level, analysis of TCGA data showed significantly increased expression of SORD in both lung adenocarcinoma and squamous cell carcinoma patients compared to normal tissues (Supplementary Fig. 1E). Protein expression data confirmed the ubiquitous occurrence of both PP enzymes in a panel of established human NSCLC cell lines derived from tumors of different histological types and mutation status (Fig. 1C, Supplementary Table 1). To decipher the impact of PP gene expression on lung tumorigenesis, we performed stable shRNA-mediated knockdown of the rate-limiting enzyme AKR1B1 using three independent sequences in various NSCLC cells (Fig. 1D). Reduction of AKR1B1 gene expression significantly decreased the metabolic flux from glucose to fructose in low AKR1B1-expressing H1299 cells as revealed by stable isotope tracing of fully labeled ^13^C-glucose (Fig. 1E and Supplementary Fig. 1F). Importantly, AKR1B1 knockdown potently halted cell proliferation in all tested cell lines (Fig. 1F and Supplementary Fig. 1G). In addition, induction of cell death in response to AKR1B1 depletion in various NSCLC cell lines was confirmed with a fluorescent dye (Fig. 1G, Supplementary Fig. 1H). Cell death was accompanied by a strong increase in γH2AX signal, which is an indication of DNA damage (Fig. 1H). The type of cell death was further refined as caspase 3/7-dependent apoptosis (Fig. 1I). Of note, low AKR1B1-expressing H1299 cells also showed significantly high growth defects in response to the knockdown (Fig. 1F, center), suggesting a requirement for baseline AKR1B1 expression levels to sustain proliferation. In contrast, stable AKR1B1 overexpression failed to accelerate cell growth (Supplementary Fig. 1I), reaffirming its basal role in NSCLC growth maintenance rather than enhancing proliferative capabilities.

**Fig. 2:**
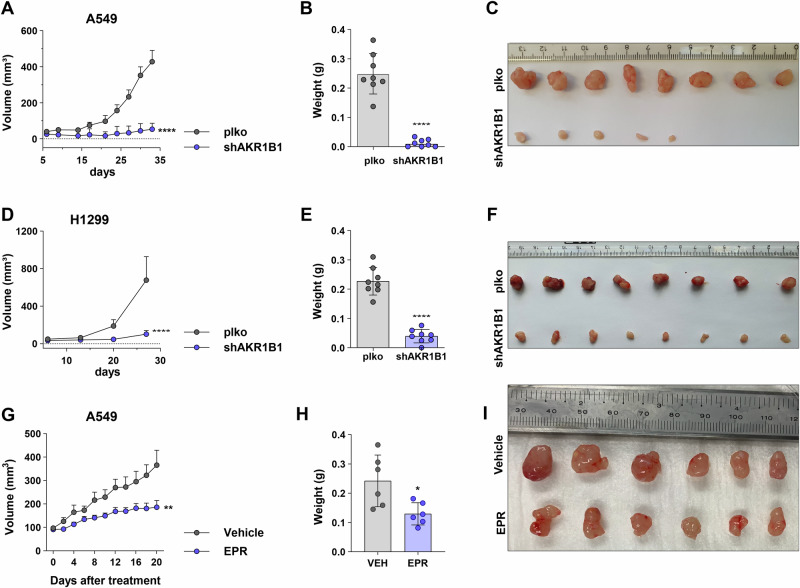
Genetic ablation of AKR1B1 attenuates the in vivo growth of NSCLC tumors. **A** Tumor growth curve showing tumor volume from NSG mice subcutaneously injected with either A549 plko control or shAKR1B1 cells as determined by caliper measurement, (**B**) weight of the excised A549 tumors and (**C**) representative image of tumors. **D** Tumor growth curve showing tumor volume from NSG mice subcutaneously injected with either H1299 plko control or shAKR1B1 cell as determined by caliper measurement, (**E**) weight of excited H1299 tumors and (**F**) representative images of tumors. **G**–**I** Tumor growth curve showing tumor volume of A549 subcutaneous xenografts treated with either epalrestat (EPR) or drug vehicle, along with the weights of the tumors and representative images. Tumor growth was analyzed using multiple *t*-tests (Holm–Sidak method) and comparison of tumor weights at endpoint was done using unpaired Student *t*-test.

We subsequently investigated in vivo tumor growth via subcutaneous injection of either control or AKR1B1 knockdown A549 cells into NSG mice. As a result, we found that reduced AKR1B1 expression remarkably impeded the ability of the cells to grow in vivo (Fig. 2A). Along with highly significant decrease in tumor mass (Fig. 2B), only 5 out of 8 injected mice formed tumors, indicating a reduction in tumor occurrence (Fig. 2C). We observed a similar trend with H1299 cells when injected in NSG mice after AKR1B1 knockdown (Fig. 2D–F). The use of epalrestat (EPR), an FDA-approved AKR1B1 inhibitor [14], was also able to reduce tumor growth in vivo in NSG mice (Fig. 2G–I).

**Fig. 3:**
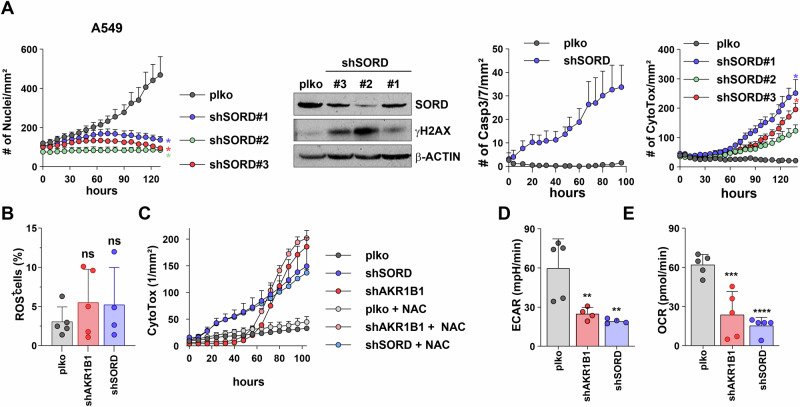
Knockdown of SORD phenocopies the growth inhibitory effect of AKR1B1 knockdown. **A** Real-time proliferation of plko and shSORD A549 cells, along with a Western blot of SORD and the DNA damage marker γH2AX, and measurement of caspase 3/7-mediated apoptosis and shSORD-induced cytotoxicity by real-time imaging. shSORD cells were generated using three independent shRNA sequences. **B** Percentage of ROS-positive A549 plko, shAKR1B1 and shSORD cells using CM-H2DCFDA as a general oxidative stress indicator. **C** Cytotoxicity assay in A549 plko control, AKR1B1 and SORD knockdown cells either in the absence or presence of 5 mM NAC. **D** Basal glycolytic activity of A549 plko, shAKR1B1 and shSORD cells as measured by the extracellular acidification rate (ECAR) upon glucose infusion during a glycolytic stress test (GST). **E** ATP-linked respiratory capacities of A549 plko, shAKR1B1 and shSORD cells as measured by the oxygen consumption rates (OCR) during a mitochondrial stress test. Statistical test in (**A**) and (**C**) is two-way ANOVA, Dunnett’s method and in (**B**, **D**, **E**) is one-way ANOVA, Dunnett’s method.

To analyze the polyol pathway activity as a whole, we extended the analysis to SORD knockdown, which is the second PP enzyme. To this end, we performed similar shRNA-mediated knockdown experiments with three independent targeting sequences in A549 cells. Similar to what we observed in shAKR1B1 cells, the downregulation of SORD gene expression severely arrested cell proliferation and induced DNA damage and apoptosis (Fig. 3A). Comparable data were obtained with the high AKR1B1-expressing NSCLC cell line, Calu-1 (Supplementary Fig. 2A) as well as cell lines with high SORD and low AKR1B1 expression, H460 and H520 (Supplementary Fig. 2B, C), indicating that both enzymes of the PP are important for proliferation. Overall, these data provide strong rationale for the polyol pathway as supportive of tumor growth and survival.

### PP deficiency causes a multifactorial phenotype that impairs cellular metabolism

As oxidoreductases are involved in antioxidant processes [15, 16], we suspected an increased ROS production as one of the causes for the detected DNA damage in polyol pathway-deficient cells (Figs. 1H and 3A). Surprisingly, no major changes in intracellular levels of ROS could be detected upon either AKR1B1 or SORD knockdown (Fig. 3B). Concomitantly, shPP-induced detrimental defects were irreversible in the presence of the ROS scavenger (N-Acetyl cysteine) NAC (Fig. 3C), indicating that the cell death induced by the knockdown of PP is not mediated via ROS accumulation. Then, analyzing their metabolic state, we found that cells deficient in either AKR1B1 or SORD expression displayed reduced glycolysis as well as oxidative phosphorylation (Fig. 3D, E). Since both pathways are major producers of ATP, and ATP deficiency might lead to cell death, we hypothesized that shPP-induced cell death could be rescued by ATP supplementation. However, we did not observe any growth rescue of shPP cells upon ATP supplementation (Supplementary Fig. 2D), indicating that ATP deficiency is not the cause of shPP-induced apoptosis. Furthermore, as induction of apoptosis in PP-deficient cells might be due to the combined effect of ROS accumulation and ATP deficiency, we also attempted to rescue shPP cells by combining NAC and ATP supplementation, but the combination did not protect the cells either (Supplementary Fig. 2E). Fructose metabolism also induces de novo lipogenesis, which was previously shown to promote lung cancer growth [17]. However, rescue experiments via fatty acids supplementation with palmitate (PA) failed to alleviate the shPP-induced defects (Supplementary Fig. 2F), hinting towards a multifactorial metabolic vulnerability.

At the molecular level, AKT is an important regulator of cell proliferation, survival as well as metabolic processes [18, 19], and has been shown to be regulated by AKR1B1 [20]. Therefore, we monitored the expression of AKT in PP-deficient cells and observed that PP downregulation in A549 cells was accompanied by a decrease in AKT1 protein levels (Supplementary Fig. 2G). However, AKT1 overexpression in PP-deficient cells failed to decrease cell death and to rescue cell growth (Supplementary Fig. 2H–J).

### Fructose displays anti-apoptotic properties

**Fig. 4:**
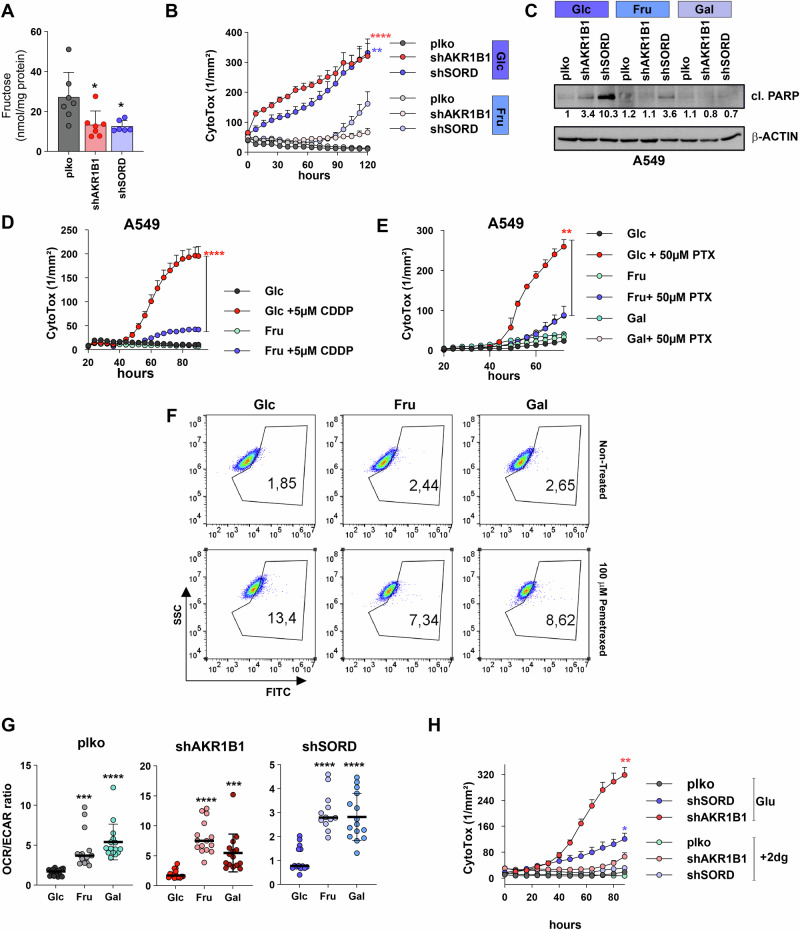
Fructose rescues cell death in polyol pathway-deficient NSCLC cells in the absence or presence of chemotherapy. **A** Mass spectrometric quantification of intracellular levels of fructose in plko and shPP A549 cells. Supplementary Fig. 3A shows glucose and fructose peak chromatographic resolution. **B** Cytotoxicity assay upon PP knockdown in A549 cells grown in glucose-free DMEM supplemented with either 5 mM glucose or fructose. **C** Western blot of cleaved PARP (cl.PARP) in plko control or PP knockdown A549 cells grown in 5 mM glucose, fructose or galactose. **D** Cytotoxicity assay upon treatment with 5 µM Cisplatin (CDDP) in A549 cells grown in glucose or fructose. **E** Cytotoxicity assay upon treatment with 50 µM Pemetrexed (PTX) in A549 cells grown in glucose, fructose or galactose (5 mM each). **F** ROS levels, indicated as the percentage of ROS^+^ population in A549 cells treated with PTX in combination with glucose, fructose or galactose supplementation as measured by flow cytometry. **G** OCR/ECAR ratio of A549 plko control and shPP cells grown in glucose, fructose or galactose as quantified by Seahorse Assay. The individual OCR and ECAR plots are shown in Supplementary Fig. 3G. **H** Cytotoxicity assay upon either treatment with sublethal doses of 2-DG (200 µM) or supplementation with glucose in A549 plko control and shPP cells. Statistical test in (**A**, **G**) is unpaired *T*-test and in (**B**, **D**, **E**, **H**) is two-way ANOVA and Tukey’s post-test for multiple comparisons.

As the only de novo pathway for fructose synthesis is PP, we observed by chromatography–mass spectrometry (GC-MS) a significant reduction of intracellular fructose levels in cells deficient in both PP enzymes (Fig. 4A, Supplementary Fig. 3A). We subsequently attempted to reverse the PP knockdown-related phenotype via supplementation with extracellular fructose. For this purpose, A549 cells were first conditioned in either glucose- or fructose-containing media for 4 days prior to knockdown induction. As indicated in Fig. 4B, the availability of fructose prevented the induction of cell death in response to AKR1B1 knockdown, while a strong delay was apparent in shSORD cells as compared to cells grown in glucose-rich conditions. The fructose-mediated death response delay was also observed in other NSCLC cells with PP gene knockdown (Supplementary Fig. 3B). Surprisingly, we also identified galactose as an alternative monosaccharide to glucose, which alleviated cell death induction upon PP knockdown (Supplementary Fig. 3C). Indeed, similar to what was observed under fructose supplementation, shAKR1B1 and shSORD cells conditioned in galactose were largely protected from cell death induction. Supporting the differential death response, we detected clear differences in the expression of cleaved poly (ADP-ribose) polymerase (PARP), a target of caspase 3 and therefore a hallmark of apoptosis. Western blot quantification revealed a strong accumulation of cleaved PARP in shAKR1B1 and shSORD cells grown in glucose-containing media, which was diminished or completely abrogated under either fructose or galactose conditions (Fig. 4C).

Moreover, cytoprotective effects of fructose and galactose were also reflected in an increased in vitro resistance to chemotherapeutic agents. Several lung cancer cell lines conditioned in glucose, fructose or galactose-containing media were subsequently exposed to either cisplatin (CDDP) [21] or pemetrexed (PTX) [22], two commonly used drugs in the treatment of advanced NSCLC. Cytotoxicity assays revealed that fructose protected against CDDP as well as PTX-induced cell death in contrast to glucose (Fig. 4D, E, Supplementary Fig. 3D). Protection against apoptosis was observed in combination with a reduction in associated ROS levels (Fig. 4F, Supplementary Fig. 3E). Galactose showed mainly protective effects associated with ROS levels reduction against PTX-triggered cytotoxicity (Fig. 4E, Supplementary Fig. 3E, F). These results point towards a general role for alternative monosaccharides in protecting against cytotoxic triggers.

These results prompted to search for commonly shared metabolic mechanisms between fructose and galactose that are distinct from glucose. Several publications have indicated a preference for oxidative phosphorylation as their favored energy-producing pathways [23, 24]. Indeed, when measuring the OCR/ECAR ratio as a readout of metabolic activity, control cells under fructose/galactose supplementation displayed significantly increased values as compared to cells supplemented with glucose (Fig. 4G, Supplementary Fig. 3G), indicating a metabolic preference for oxidative phosphorylation. Similarly, fructose/galactose-supplemented shAKR1B1 or shSORD cells also increased their oxidative phenotype as compared to their glucose counterparts (Fig. 4G). Therefore, this metabolic shift might indicate that a more oxidative metabolic phenotype underlies the protective effect of fructose and galactose in PP-deficient cells. To test whether this metabolic shift is protecting against shPP-mediated cell death, we used 2-deoxy-d-glucose (2-DG), a synthetic glucose analog and well-described glycolysis inhibitor, to force cells to switch from glycolysis to oxidative phosphorylation [25, 26]. PP knockdown experiments were performed either in the presence or absence of low concentrations of 2-DG, which did not affect the in vitro proliferation of A549 cells (Supplementary Fig. 3H). In line with the effect observed with alternative sugars, 2-DG did not improve the proliferative ability of PP knockdown cells, but remarkably prevented (Fig. 4H) or diminished (Supplementary Fig. 3I) shPP-induced cytotoxicity in different lung cancer models. In conclusion, these results suggest the pro-survival properties of fructose and galactose in PP-deficient cells might be partly executed via oxidative phosphorylation.

### RNA sequencing reveals differential death response between glucose and other hexoses

**Fig. 5:**
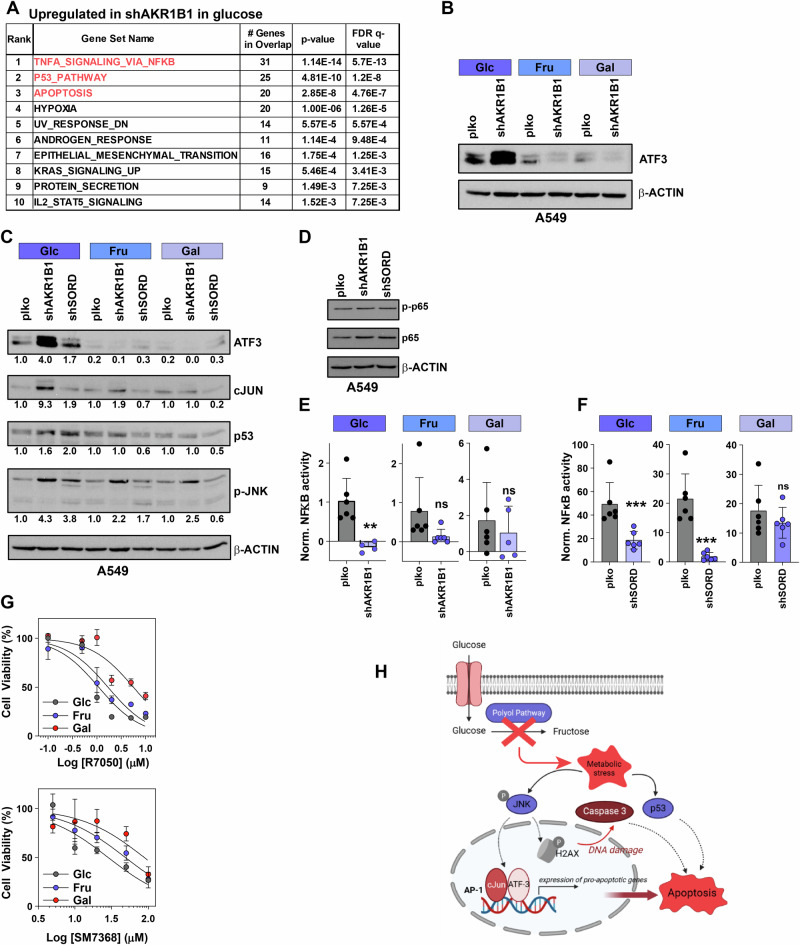
Anti-apoptotic properties are associated with fructose metabolism in NSCLC cells. **A** Gene Set Enrichment Analysis showing upregulated pathways in AKR1B1 knockdown cells as compared to plko control cells under glucose supplementation. **B** Western blot of ATF3 in A549 plko control and shAKR1B1 cells grown in glucose, fructose or galactose (5 mM each). **C** Western blot of ATF3, c-JUN, p53 and phospho-JNK in A549 plko control and shPP cells grown in glucose, fructose or galactose. **D** Western blot of total and activated phosphorylated p65 (p-p65) in A549 plko control and shPP cells. **E** NF-κB activity luciferase reporter assay in A549 plko control and shAKR1B1 cells grown in glucose, fructose or galactose. **F** NF-κB activity luciferase reporter assay in A549 plko control and shSORD cells grown in glucose, fructose or galactose. **G** Cell viability assay in A549 cells grown in glucose, fructose or galactose upon treatment with increasing concentrations of NF-κB inhibitors R7050 (top) or SM7368 (bottom). Inhibition response curves were plotted by determining the confluency of control and treated group after 40 h of treatment, normalized to controls, set at 1, and subsequent log transformation of drug concentrations. **H** A schematic representation of the potential role of the polyol pathway in cancer cell survival. The model suggests that deficit of fructose due to disruption of the polyol pathway (PP) triggers a metabolic stress that leads to activation of the JNK/c-Jun pathway and ATF3 resulting in activation of the pro-apoptotic AP-1 complex in parallel with a potential activation of p53. Statistical test in (**F**) is unpaired *t*-test and Dunnett’s method for multiple comparisons.

To decipher molecular mechanisms associated with the differential death response under the presence of different monosaccharides, RNA sequencing was performed on shAKR1B1 and control cells, maintained under either glucose or fructose-conditioned media. Hierarchical cluster analysis and principal component analysis indicated a clear separation between control and knockdown samples and based on the availability of glucose or fructose in the media (Supplementary Fig. 4A, B). Differential gene expression analysis revealed an overlap of 38.3% identical genes, which were commonly downregulated in shAKR1B1 cells under both glucose and fructose supplementation (Supplementary Fig. 4C). Gene set enrichment analysis (GSEA) indicated that this list of genes was most significantly represented in cell cycle pathways, E2F targets and G2M checkpoints (Supplementary Fig. 4D), corroborating the shared proliferative deficits observed in both glucose- and fructose-grown AKR1B1 knockdown cells. More importantly, the 1415 upregulated genes in shAKR1B1 cells under glucose supplementation were most significantly classified in pathways related to apoptosis, tumor necrosis factor alpha (TNF-α)- and p53 signaling (Fig. 5A), confirming the strong cell death induction observed in response to AKR1B1 deficit. In contrast, genes upregulated in fructose-grown shAKR1B1 cells were only enriched for a hypoxia gene signature, and with a low q-value (Supplementary Fig. 4E). The lack of clear apoptosis-related gene signatures under fructose conditions corroborates the differential death response observed in vitro (Fig. 4B). This is also reaffirmed by comparing gene sets downregulated in shAKR1B1-fructose cells compared to glucose counterparts, which revealed the apoptosis-related pathways to be the most significantly enriched (Supplementary Fig. 4F). This indicates that the differential cell death signature is the strongest discriminant between glucose- and fructose-grown AKR1B1 knockdown cells.

Among the genes most significantly and exclusively upregulated in glucose-grown shAKR1B1 cells, two candidates, ATF3 and c-Jun were selected for further validation. Selection was based on the involvement of these two genes in all the three signatures for ‘apoptosis’, ‘p53 pathway’ and ‘TNF-α signaling’. First, protein expression of ATF3, an apoptotic marker, was found markedly upregulated in shAKR1B1 cells under glucose supplementation, but neither under fructose nor galactose (Fig. 5B). Rather, no differences and even downregulation of ATF3 expression were observed in shAKR1B1 cells under fructose and galactose as compared to controls. This analysis was further extended to shSORD cells for all growth conditions (Fig. 5C). Here, ATF3 levels were also increased under glucose supplementation, and unchanged as compared to their respective controls in the fructose and glucose counterparts.

The other candidate examined was the transcription factor c-Jun, whose expression was also stimulated in response to AKR1B1 depletion under glucose supplementation. c-Jun upregulation occurred to a lesser extent upon SORD depletion under glucose supplementation (Fig. 5C). In knockdown cells under fructose or galactose supplementation, c-Jun increase was either attenuated or completely absent (Fig. 5C).

Both c-Jun and its transcriptional target ATF3 [27] exercise various functions regarding cell fate decisions and are known to play a role in apoptosis activation under specific conditions in connection with p53 [28, 29]. Interestingly, we also identified differences in p53 expression. Deficiency of both AKR1B1 and SORD increased p53 protein levels under glucose, but neither under fructose nor galactose conditions (Fig. 5C). In addition, we also observed activation of JNK (c-Jun N-terminal kinase) in response to PP deficiency under glucose, which is indicated by its increased phosphorylation status (p-JNK) (Fig. 5C). As JNK is a known stress-activated kinase with pro-apoptotic functions, higher activation by phosphorylation was not seen in both PP knockdown cells under either fructose or galactose supplementation, although to a lesser extent compared to c-Jun. We therefore propose that depletion of both PP genes activates apoptosis over a common signal transduction pathway involving JNK/c-Jun in cooperation with p53. The lack of increased apoptosis-related proteins under the presence of fructose or galactose might indicate a commonly shared pathway, which enables PP knockdown cells to prevent this activation and ultimately develop resistance towards apoptosis.

These results prompted us to investigate possible pro-survival mechanisms. Interestingly, AKR1B1 silencing has been previously connected to decreased NF-κB activity [11, 30], a pathway commonly involved in cell survival [31]. We, indeed, observed a significantly high enrichment of NF-κB pathway in shAKR1B1 cells by RNA sequencing (Fig. 5A). Therefore, we sought to investigate the involvement of the NF-κB pathway in shPP-induced cell defects and differential death responses. First, we analyzed whole protein and phosphorylation levels of p65/RelA, an important NF-κB subunit, but were unable to detect changes upon AKR1B1 or SORD depletion (Fig. 5D). In contrast, shAKR1B1 and shSORD cells in the presence of glucose displayed a strongly reduced NF-κB activity in a luciferase reporter assay, whereas the activity especially under the presence of galactose was not significantly changed **(**Fig. 5E, F). Based on this result, we tested the cell dependency on active NF-κB signaling by treating A549 cells grown in glucose, fructose or galactose with increasing concentrations of two NF-κB inhibitors. Inhibition curves revealed that galactose and, to a lesser extent fructose confer resistance against NF-κB inhibition (Fig. 5G). In conclusion, latest results could indicate that these monosaccharide substitutes may confer survival benefits and drug resistance by maintaining NF-κB activity.

## Discussion

This study provides evidence for the importance of the polyol pathway activity in conferring growth and survival advantages to lung cancer cells. Contrary to the widespread claim that this pathway has little activity under normal physiological conditions [6], the detrimental effects observed upon its downregulation, as evidenced by dramatic growth defects in vitro and in vivo (Fig. 1), reveal an essential role of the PP in maintaining normal cellular functions. While the inflammatory function of AKR1B1 in tumorigenesis has been previously described [11], this research highlights the critical role of SORD activity and therefore the functionality of both PP genes as indispensable for survival and proliferation homeostasis. The complexity of the multifaceted phenotype is further reflected in the inability to restore cellular proliferation by means of individual biomolecule substitution as well as combinations. However, fructose-mediated cell survival could take different molecular and biochemical routes or a combination of both factors, and therefore, a dedicated study is needed determine the downstream effectors of the polyol pathway. Considering the negligible pathway activity under normoglycemic conditions in healthy tissues, targeting the cancer specific PP activity seems ideally suited for therapeutical intervention. In general, the ineffectiveness of various proliferation rescue approaches, either through restoring oncogenic signaling or macromolecules supplementation, provides major therapy advantages, as it could potentially complicate the emergence of therapy resistance.

At a molecular level, the cytotoxic phenotype induced by the reduction of PP activity was associated with upregulation of ATF3 and c-Jun, transcription factors with well-described pro-apoptotic functions [28, 29, 32]. However, given the complexity and context-dependency of the AP-1 transcription factor family, further investigation is necessary to determine whether the ATF3/c-Jun complex activation is directly caused by PP deficiency.

Importantly, we uncovered an important role for hexose sugar replacements in resisting cell death. Therefore, with this study we attribute a pro-survival function of PP-derived fructose as one of its main roles in the context of lung cancer. We demonstrate that cells under the presence of fructose are able to suppress pro-apoptotic signaling as demonstrated by the lack of Caspase 3 activation. Interestingly, previous publications have reported on cytoprotective properties of fructose [33] as well as other sugar hexoses, such as galactose [34] against TNF-α-induced apoptosis. More specifically, fructose-associated protection was mediated via suppression of JNK signaling [33], whereas galactose prevented cell death through NF-κB activation [34]. We, indeed, provide evidence for both of them in this study. One of the best-described functions of fructose metabolism is the induction of de novo lipogenesis, which was previously shown to promote lung cancer growth [17, 35]. However, supplementation with fatty acids, such as palmitate, to circumvent a potential reduction of fatty acid synthesis, did not ameliorate the phenotype induced by PP knockdown. This suggests that the fructose-associated lipid metabolism might be dispensable for preventing shPP-induced cell death. One possible mechanism through which glucose alternatives could overcome shPP-related vulnerabilities could be via a metabolic shift of cells to oxidative phosphorylation, well described under the presence of galactose [23] and fructose [36], in particular for the later. However, although an oxidative phenotype has been associated with survival benefits in various cancer types [37], further research is needed to confirm a direct contribution and the molecular determinants of alternative metabolic preferences to the apoptosis-resistance phenotypes displayed upon fructose and galactose availability. Their ability to withstand cell death has widespread implications not only in the context of PP activity, but for chemotherapy resistance (Fig. 4). Roles of fructose metabolism in cancer should be especially studied considering the massive increase of dietary fructose intake and its well-established association with obesity, cardiovascular disease and cancer [38]. Therefore, the results presented in this paper broaden our understanding of fructose metabolism in cancer growth, in part as a pathway that may offer sufficient metabolic plasticity to cancer cells and, thus, facilitate tumor survival (Fig. 5H).

## Supplementary information

Western Blot

Supplementary Material

## Data Availability

RNA sequencing data are deposited in the GEO database with the accession number GSE214505.
