# Supplementary material for: Polyol pathway-generated fructose is indispensable for growth and survival of non-small cell lung cancer
Source: Cell Death Differ. 2024 Nov 20;32(4):587–97. doi: 10.1038/s41418-024-01415-1 (PMC11982217; doi:10.1038/s41418-024-01415-1)

Figure 1C

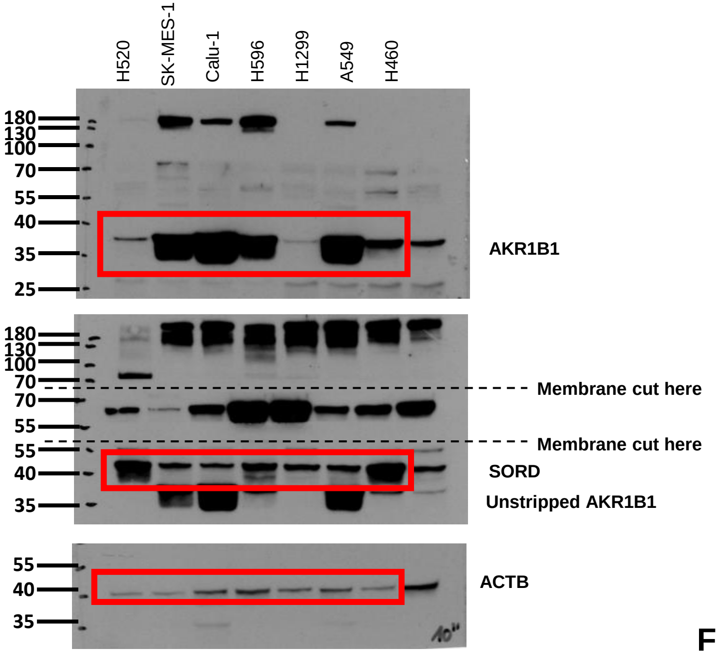

Non-small Cell Lung Cancer

Figure 1D:A549

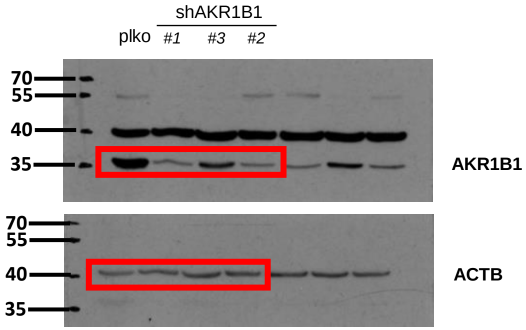

Figure 1D:H1299

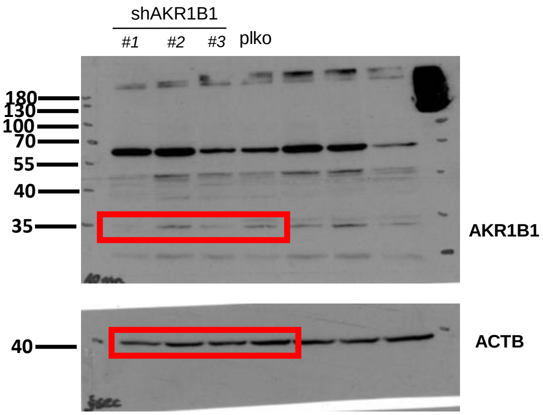

Note: The blot was flipped in the manuscript

Figure 1D:Calu1

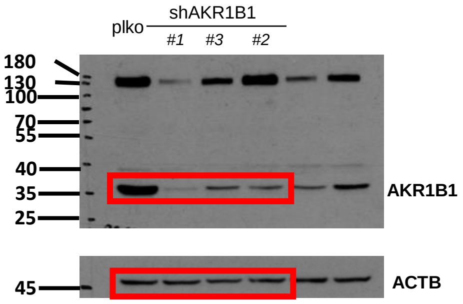

Figure 1H

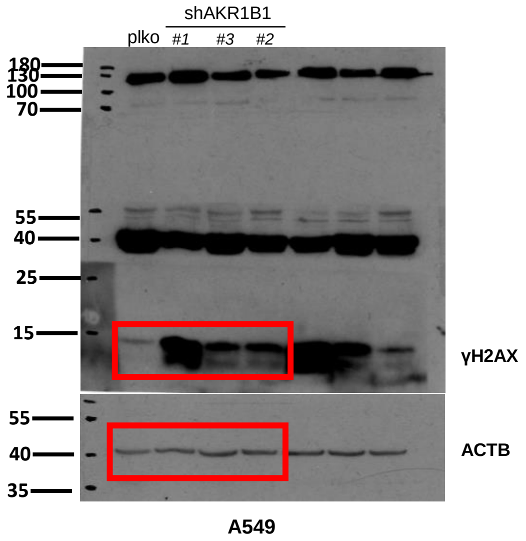
shAKR1B1
plko
#1
#3
#2
180
130
100
70
55
40
25
15
γH2AX
55
40
35
ACTB
A549

Figure 3A

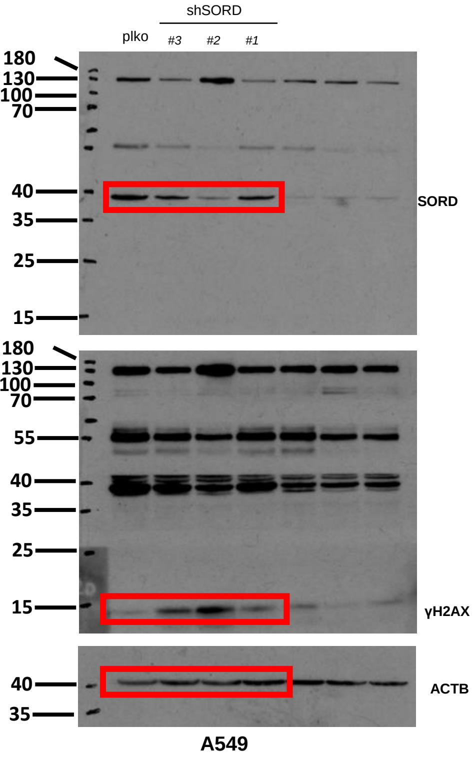
shSORD
plko
#3
#2
#1
180
130
100
70
40
35
25
15
SORD
180
130
100
70
55
40
35
25
15
γH2AX
40
35
ACTB
A549

Figure 4C

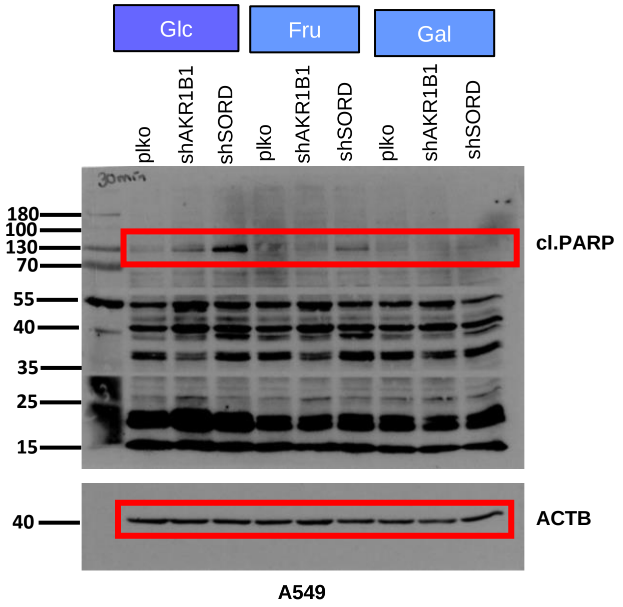

Glc
Fru
Gal
plko
shAKR1B1
shSORD
plko
shAKR1B1
shSORD
plko
shAKR1B1
shSORD
180
100
130
70
55
40
35
25
15
cl.PARP
40
ACTB
A549

Figure 5B

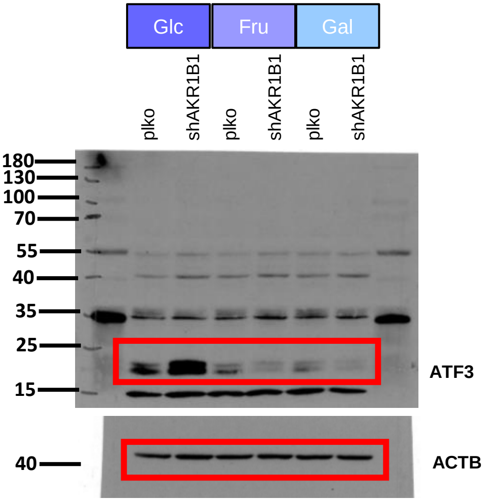

A549

Figure 5C

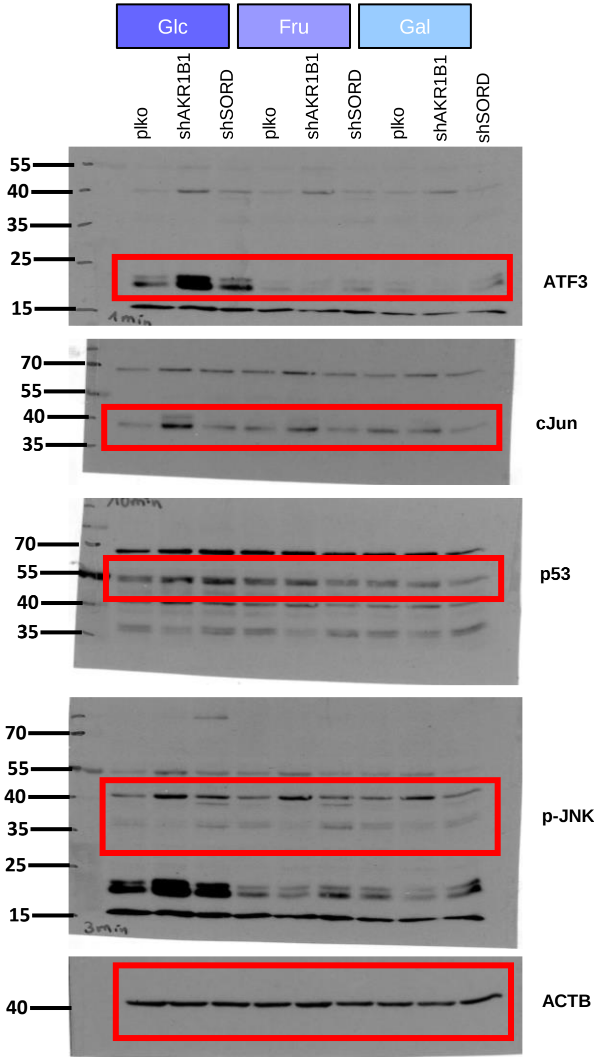

A549

Figure 5D

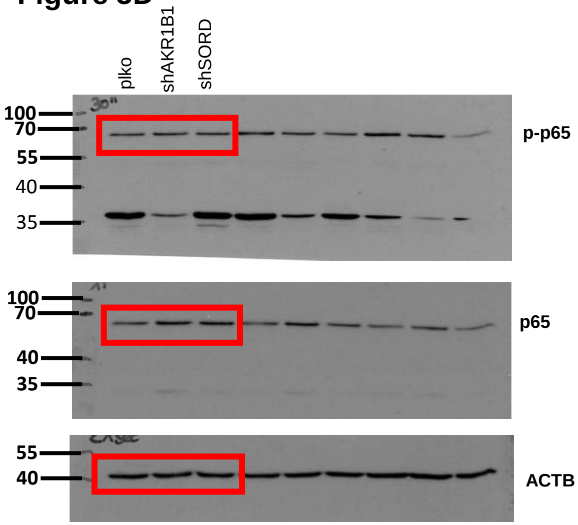

A549

Supplementary Figure 1G

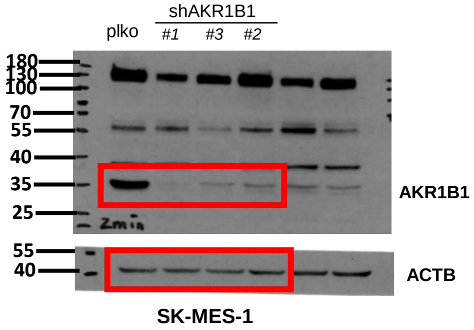

Supplementary Figure 1I

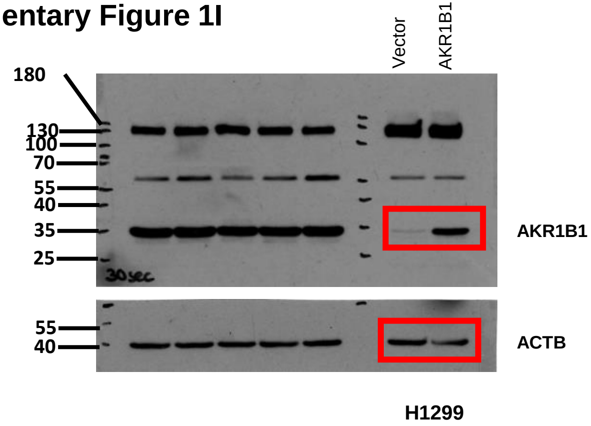

Supplementary Figure 3B

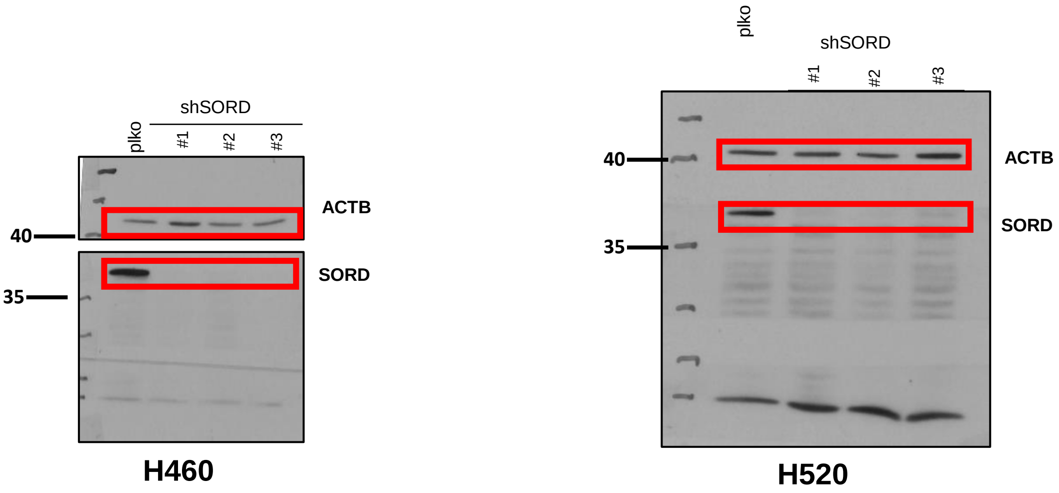

Supplementary Figure 2G

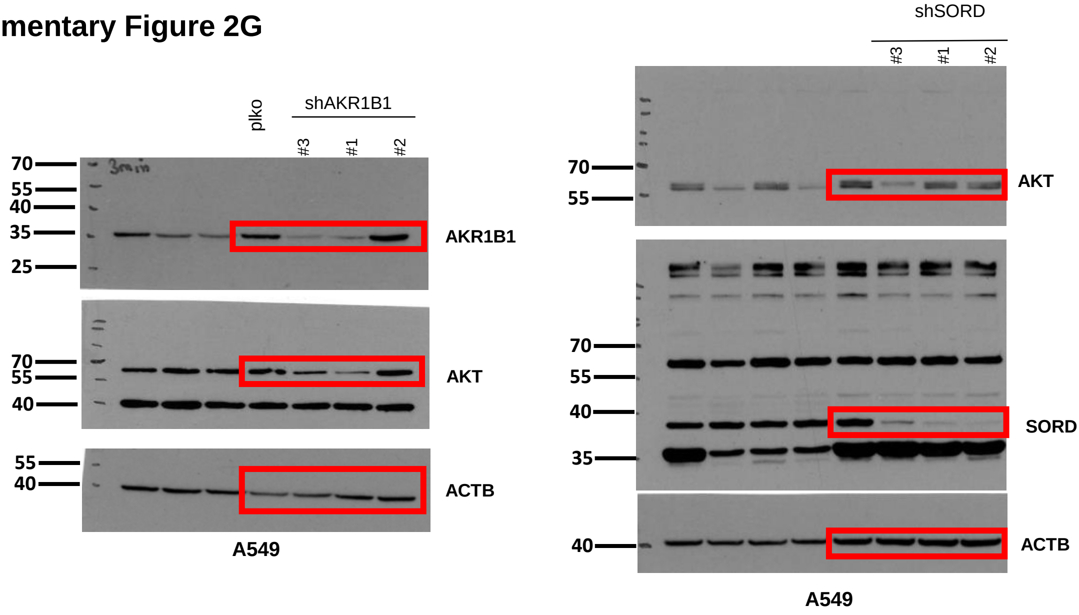

Supplementary Figure 2H

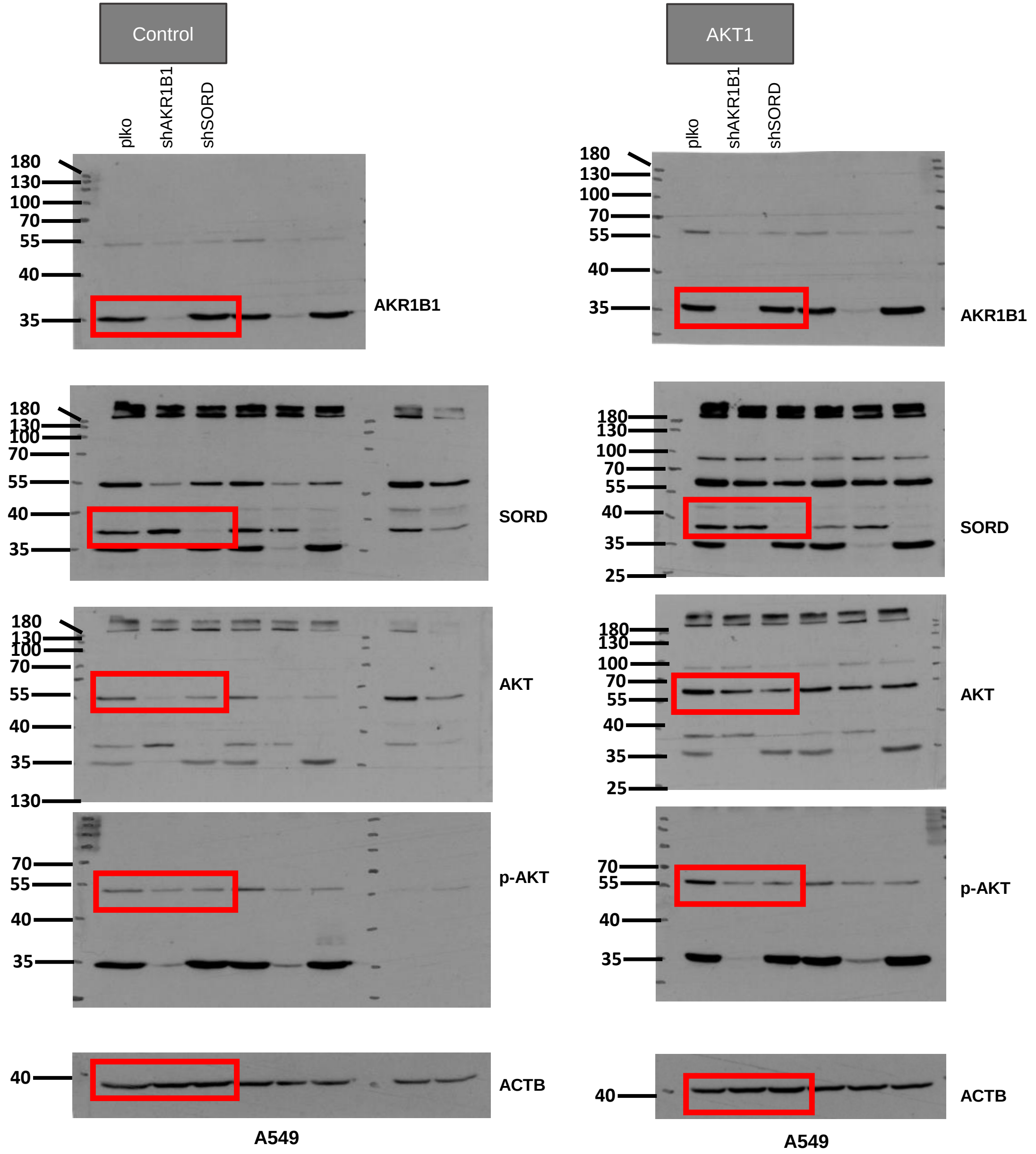

Supplement: Supplementary file 1 — Western Blot [file 41418_2024_1415_MOESM1_ESM.pdf]
