# Supplementary material for: Polyol pathway-generated fructose is indispensable for growth and survival of non-small cell lung cancer
Source: Cell Death Differ. 2024 Nov 20;32(4):587–97. doi: 10.1038/s41418-024-01415-1 (PMC11982217; doi:10.1038/s41418-024-01415-1)
Supplement: Supplementary file 2 — Supplementary Material [file 41418_2024_1415_MOESM2_ESM.pdf]

Supplementary Fig. 1

A

Mean Patient Age	*71 years*
**Gender**	
Male	*40*
Female	*49*
**Stage**	
I	*56*
II	*17*
III	*16*
**Histopathology**	
Adenocarcinoma	*53*
Sqamous Cell Carcinoma	*27*
Others	*9*

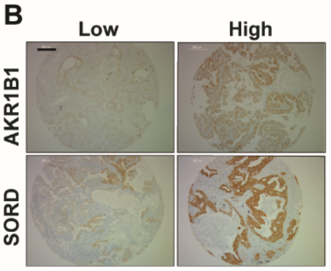

C

		LUAD	LUSC	Others
AKR1B1	Pos	*20*	*8*	*4*
	Neg	*33*	*19*	*5*
SORD	Pos	*38*	*15*	*4*
	Neg	*15*	*12*	*5*

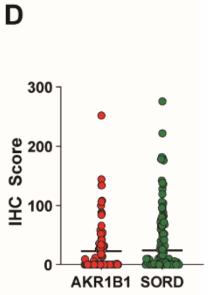

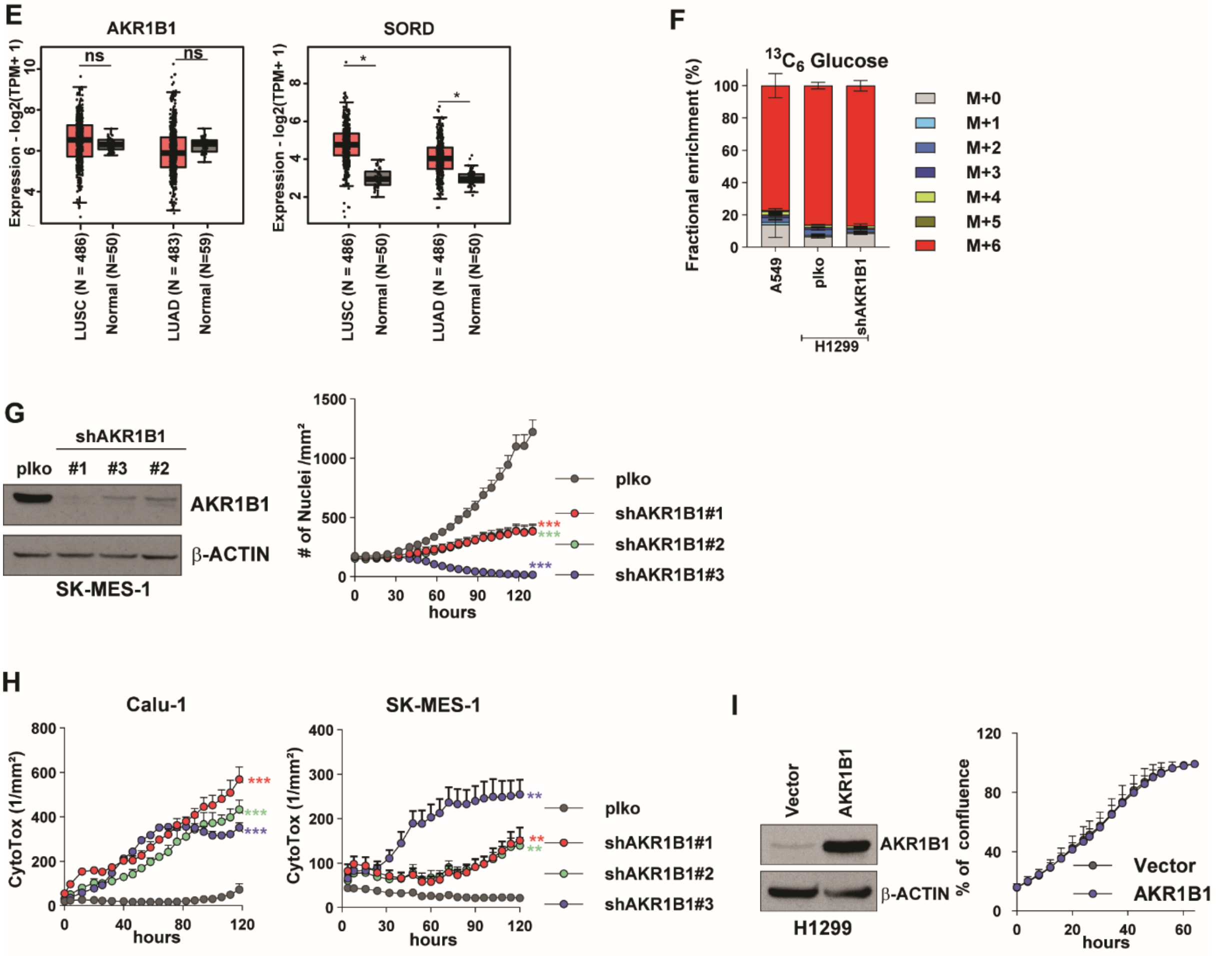

Supplementary Figure 2

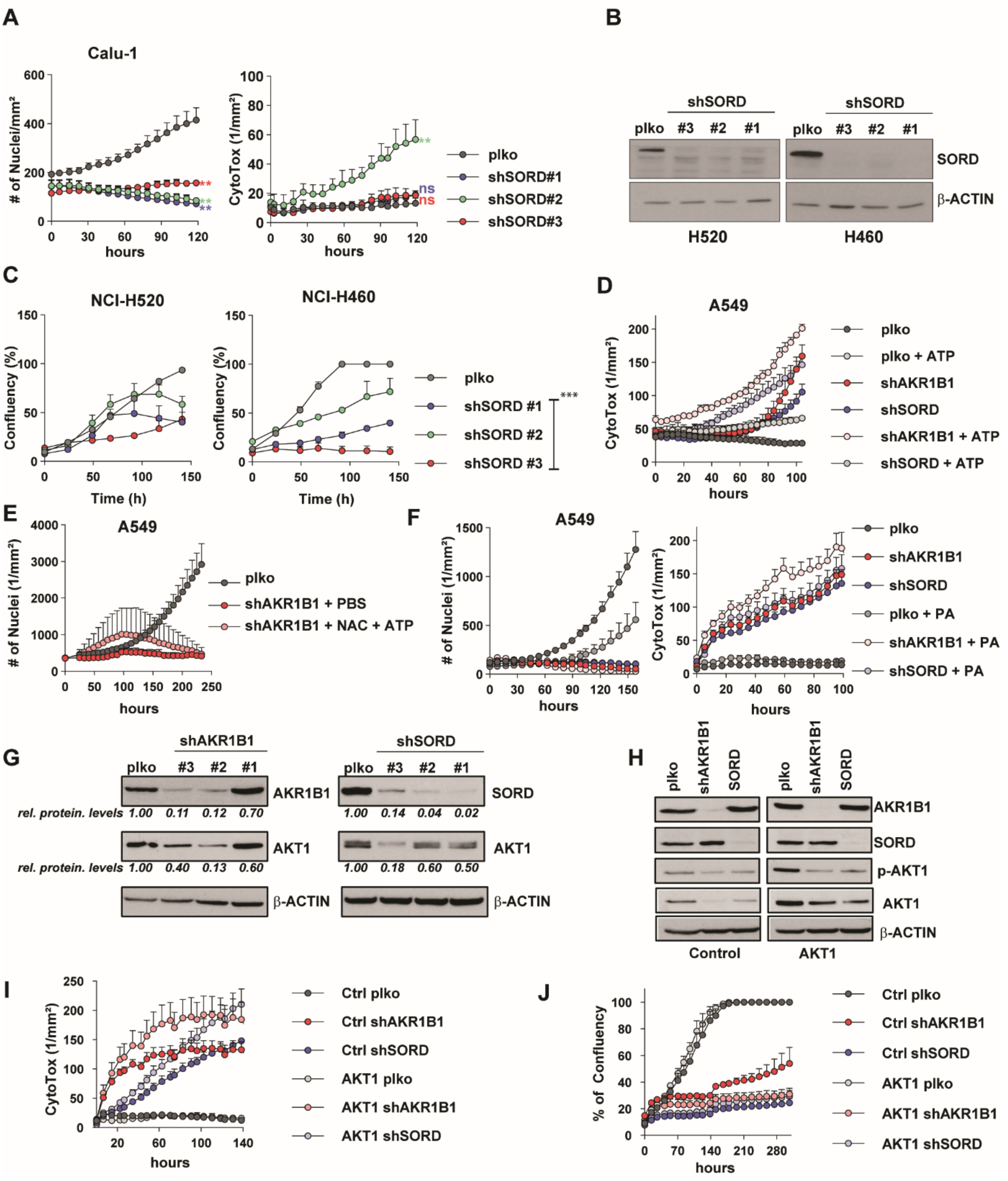

Supplementary Figure 3

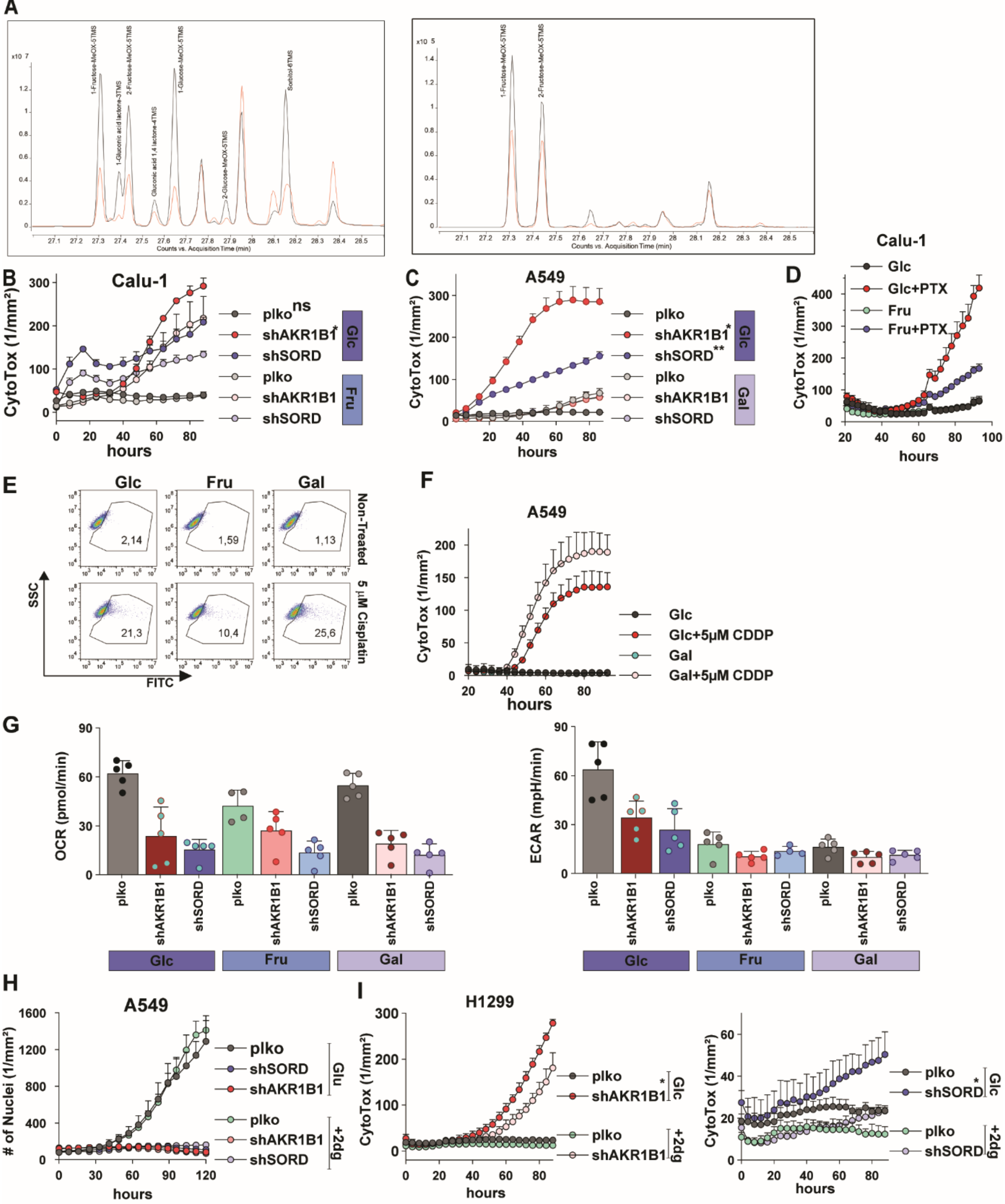

Supplementary Figure 4

A

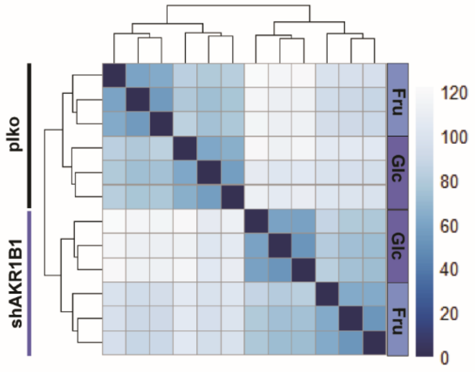

B

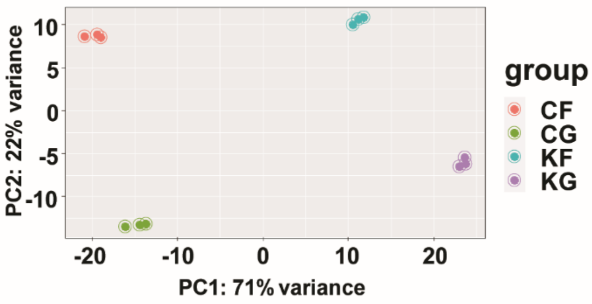

C

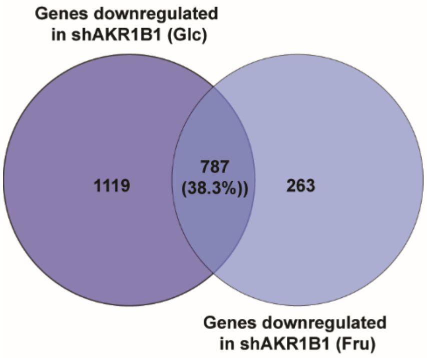

D Commonly donwregulated in shAKR1B1 in fructose and glucose

Rank	Gene Set Name	# Genes in Overlap	p-value	FDR q-value
1	REACTOME_CELL_CYCLE	82	2.43E-40	4.38E-37
2	HALLMARK_E2F_TARGETS	45	7.21E-35	6.5E-32
3	REACTOME_CELL_CYCLE_MITOTIC	65	1.5E-31	9.01E-29
4	HALLMARK_G2M_CHECKPOINT	42	2.61E-31	1.18E-28
5	REACTOME_CELL_CYCLE_CHECKPOINTS	45	2.11E-27	7.62E-25
6	REACTOME_RESOLUTION_OF_SISTER_CHROMATID_COHESION	25	1.54E-18	4.65E-16
7	REACTOME_MITOTIC_PROMETAPHASE	30	3.57E-18	9.22E-16
8	REACTOME_MITOTIC_SPINDLE_CHECKPOINT	23	1.31E-17	2.96E-15
9	KEGG_CELL_CYCLE	24	1.67E-17	3.35E-15
10	REACTOME_RHO_GTPASES_ACTIVATE_FORMINS	23	2.79E-15	5.04E-13

E Upregulated in shAKR1B1 in fructose

Rank	Gene Set Name	# Genes in Overlap	p-value	FDR q-value
1	HALLMARK_HYPOXIA	10	5.66E-4	2.83E-2

F Downregulated in shAKR1B1 in fructose

Rank	Gene Set Name	# Genes in Overlap	p-value	FDR q-value
1	TNFA_SIGNALING_VIA_NFKB	16	2.61E-16	1.31E-14
2	APOPTOSIS	9	2.43E-8	6.07E-7
3	P53_PATHWAY	7	2,00E-05	3.33E-4
4	UV_RESPONSE_UP	6	5.04E-5	6.3E-4
5	KRAS_SIGNALING_UP	5	1.44E-3	1.44E-2
6	HYPOXIA	4	9.42E-3	7.85E-2

Legends for Supplementary Figures

Supplementary Figure 1.

(A) Table of the characteristics of the patients analyzed for their expression of AKR1B1 and SORD by IHC in Fig. 1A-B**. (B)** Representative TMA samples showing high and low staining for AKR1B1 and SORD. Scale bar represents 200 µm. **(C)** Table indicating the number of AKR1B1 and SORD positive and negative cases distributed for NSCLC subtypes, lung adenocarcinoma (LUAD) and lung squamous carcinoma (LUSC) **(D)** of IHC scores of AKR1B1 and SORD from Fig 1A-B. **(E)** mRNA expression of PP genes in LUAD and LUSC compared to normal tissues. **(F)** Fractional enrichment of glucose in A549 plko cells and H1299 plko and shAKR1B1 upon [U-$^{13}C_6$]-Glucose tracing shown in Fig. 1E. P-values indicate significant differences among M+6 isotopologues **(G)** Western blot of AKR1B1 in SK-MES scrambled plko control and AKR1B1 knockdown cells transduced with three independent shRNA sequences and real-time proliferation. Proliferation of SK-MES H2B-RFP positive cells was determined via quantification of fluorescent nuclei counts over time by the real-time imaging system Incucyte. **(H)** Cytotoxic effect of shAKR1B1 in Calu-1 and SK-MES cells as quantified by the fluorescence of a green CytoTox dye in dead cells. **(I)** Western blot of AKR1B1 in H1299 control and AKR1B1-overexpressing cells and cell confluency quantified by real-time imaging. Statistical test in (E and F) is unpaired T-test and in (G and H) is two-way ANOVA and Tukey's post-test for multiple comparisons.

Supplementary Figure 2.

(A) Real-time proliferation and cytotoxic effect of Calu-1SORD knockdown cells transduced with three independent shRNA sequences. Proliferation and cytotoxicity were quantified by real-time imaging. **(B)** Western blot detecting SORD knockdown in H460 and H520 cells by three shRNA sequences. **(C)** Cell confluency of H460 and H520 with control plko and shSORD cells as quantified by real-time imaging. **(D)** Cytotoxic effect of PP knockdown in A549 either in the absence or presence of 100 µM ATP. **(E)** Real-time proliferation of A549 shPP cells in the presence of 100 µM ATP and 5 mM NAC. **(F)** Real-time proliferation and cytotoxic effect

of shPP in A549 cells in the absence or presence of palmitate (PA) supplementation. **(G)** Western blot of AKR1B1, SORD and AKT1 in A549 shPP cells and **(H)** Western blot of AKR1B1, SORD and AKT1 and phospho-AKT1 in plko control and shPP A549 cells which overexpress AKT1 or a control vector. **(I)** Cytotoxic effect of PP knockdown in A549 cells upon overexpression of AKT1. **(J)** Real-time proliferation of plko control and shPP cells upon AKT1 overexpression by real-time imaging. Statistical tests in all plots are two-way ANOVA and Tukey's post-test for multiple comparisons.

Supplementary Figure 3.

(A) Chromatograms showing elution peaks for glucose and fructose in the Fig. 4A. **(B)** Cytotoxic effects of PP knockdown in the Calu-1 cells in the presence of glucose (Glc) or fructose (Fru). **(C)** Cytotoxic effects of PP knockdown in A549 cells in the presence of glucose or galactose (Gal). **(D)** Cytotoxic effects of 100 μM pemetrexed (PTX) in presence or absence of glucose and fructose in Calu-1 cells. (**E**) ROS levels (%) in cisplatin treated A549 cells that were cultured in medium supplemented with glucose, fructose or galactose as measured by flow cytometry. **(F)** Cytotoxic effect of cisplatin in presence or absence of glucose or galactose in A549 cells. **(G)** OCR and ECAR measurements in A549 shPP cells upon supplementation with glucose, fructose or galactose. **(H)** Real-time proliferation of A549 shPP cells upon supplementation with glucose or the glycolysis inhibitor 2-DG. (**G**) Cytotoxic effect of shAKR1B1 (left) and shSORD (right) in H1299 cells upon supplementation with glucose or 2-DG.

Supplementary Figure 4.

(A) Hierarchical cluster analysis and **(B)** principal component analysis of differentially expressed genes in A549 plko and shAKR1B1 knockdown cells grown under glucose or fructose supplementation. (**C**) Overlap of differentially downregulated genes in shAKR1B1 cells grown in glucose or fructose supplementation (**D**) GSEA enriched pathways of commonly downregulated genes in shAKR1B1 cells under fructose and glucose. (**E**) Signature pathways

from the genes upregulated in fructose-grown shAKR1B1 cells. (**F**) Enriched pathways from the genes downregulated in fructose-grown shAKR1B1 cells as compared to glucose-grown shAKR1B1 cells.

Supplementary Table 1

	Histopathology	AKR1B1	SORD	Oncogenic Mutation	p53 Status	Other Tumor suppressor Mutations
**H520**	Squamous Cell Carcinoma	Low	High		Mutation	CDKN2A, ATM
**SK-MES-1**	Squamous Cell Carcinoma	High	Low	*NA*	Mutation	
**Calu-1**	Squamous Cell Carcinoma	High	Low	KRAS-*G12C*	Homozyg ous Deletion	
**H596**	Adenocarcinom a	High	Low	PIK3CA	Mutation	RB
**H1299**	Large Cell Carcinoma	Low	Low	NRAS	Homozyg ous Deletion	
**A549**	Adenocarcinom a	High	Low	KRAS, SKT11	wild type	SKT11
**H460**	Large Cell Carcinoma	Low	High	KRAS, PIK3CA	Not-reported	SKT11
Source	https://www.cellosaurus.org/					
